# SERS-Based Colloidal Aptasensors for Quantitative Determination of Influenza Virus

**DOI:** 10.3390/ijms22041842

**Published:** 2021-02-12

**Authors:** Dmitry Gribanyov, Gleb Zhdanov, Andrei Olenin, Georgii Lisichkin, Alexandra Gambaryan, Vladimir Kukushkin, Elena Zavyalova

**Affiliations:** 1 Institute of Solid State Physics of Russian Academy of Science, 142432 Chernogolovka, Russia; digrib@gmail.com; 2 Chemistry Department, Lomonosov Moscow State University, 119991 Moscow, Russia; gleb.zhdanov@chemistry.msu.ru (G.Z.); ayolenin@yandex.ru (A.O.); lisich@petrol.chem.msu.ru (G.L.); 3 Chumakov Federal Scientific Center for Research and Development of Immune and Biological Products RAS, 108819 Moscow, Russia; al.gambaryan@gmail.com

**Keywords:** aptamer, aptasensor, influenza, nanoparticles, SERS, virus detection

## Abstract

Development of sensitive techniques for rapid detection of viruses is on a high demand. Surface-enhanced Raman spectroscopy (SERS) is an appropriate tool for new techniques due to its high sensitivity. DNA aptamers are short structured oligonucleotides that can provide specificity for SERS biosensors. Existing SERS-based aptasensors for rapid virus detection had several disadvantages. Some of them lacked possibility of quantitative determination, while others had sophisticated and expensive implementation. In this paper, we provide a new approach that combines rapid specific detection and the possibility of quantitative determination of viruses using the example of influenza A virus.

## 1. Introduction

Viral pandemics cause serious damage to both public health and economics. Most viral pandemics have been caused by influenza A viruses. The most dangerous influenza pandemic was in 1918–1920 (“spanish flu”, H1N1 subtype); the latest influenza pandemic was in 2009–2010 (“swine flu”, H1N1 subtype). In 2019, a new strain of coronavirus caused a potent outbreak with relatively high percentage of deaths (“COVID-19”, SARS-CoV-2 strain). One more significant pandemic was caused by human immunodeficiency virus (HIV, mainly HIV-1 strain); this pandemic has been the most long-lasting, and it has caused the highest number of deaths among recent viral pandemics [1].

Recurrent pandemics make us face the problem of the absence of affordable, rapid and precise diagnostic tools. Polymerase chain reaction (PCR) is a contemporary “gold standard” due to high sensitivity and accuracy [2,3]. PCR can be used both for qualitative and quantitative estimation of viral genomes. However, PCR requires several hours to perform 30–40 cycles. Several attempts have been made to create rapid PCR within small portable devices; their robustness and accuracy are to be studied in the near future [4]. Antibody-based assays have been used for rapid diagnostics of influenza A, HIV and other viruses. These assays include techniques with detection of enzymatic activity, fluorescence or chemiluminescence [5,6]; most of them require an advanced laboratory setting and several hours for an execution. Among them, lateral flow immunochromatographic assays (LFIA) are rapid and are the simplest in performance necessary for practical usage, but they provide only qualitative estimations [7,8,9,10,11]. Their limit of detection (LOD) is higher by 3–4 orders compared to PCR techniques, being more than 10^6^ viral particles per mL (VP/mL) [7,8,9,10]. A number of extremely sensitive techniques have been developed, but they have not been optimized for routine usage because of long-lasting sophisticated sample preparation and/or usage of expensive high-technology equipment [3,12,13,14,15]. The development of simple, rapid and sensitive techniques for virus determination is in progress [3].

Biosensors with aptamers as recognition elements are of particular interest for virus determination due to their specificity, sensitivity and availability of aptamer functionalization with a variety of labels [3,16]. In our previous work, a SERS-based aptasensor for qualitative determination of influenza viruses had an intermediate limit of detection; the LOD was 10^4^ viral particles per sample, which is 10-fold higher compared to PCR and 100-fold lower compared to antibody-based strips [17]. Specificity was achieved due to DNA aptamer RHA0385 to hemagglutinin (HA, surface protein of influenza virus); this aptamer binds different strains of influenza A with nearly the same affinity (K_D_ = 2–5 nM [18]), providing a possibility of strain-independent detection of the virus [17]. Surface-enhanced Raman spectroscopy (SERS) provided the high sensitivity of the technique; SERS-active surfaces were created by the deposition of silver nanoparticles on the silica substrates. The overall duration of the analysis was less than 15 min. However, the dependence of an analytical signal from a viral load had a complex shape that did not allow quantitative determination of the viruses [17].

In this paper, we describe a novel SERS-based aptasensor for quantitative determination of influenza A viruses that matches the requirements for further practical implementation. Colloidal silver nanoparticles (AgNP) were used instead of solid-state substrates. Colloidal AgNP have several advantages, including the following: (1) simple and cheap one-pot synthesis without the use of high-technology equipment [19]; (2) an analytical signal that has predominantly monotonous concentration dependence [20]; (3) colloidal AgNP are surrounded by an aqueous homogenous environment that decreases heterogeneity in distribution of substances. The combination of SERS on colloidal AgNP and specific recognition of viruses by aptamers can provide a simple, cheap and rapid technique for the quantitative determination of viruses.

## 2. Materials and Methods

Inorganic salts and buffer solutions were purchased from Sigma-Aldrich (St. Louis, MO, USA) and MP Biomedicals (Illkirch-Graffenstaden, France). The following modified oligonucleotides were used: RHA0385-SH (5′-HS-(CH_2_)_6_-TTGGGGTTATTTTGGGAGGGCGGGGGTT-3′) from Synthol (Moscow, Russia) and RHA0385-BDPFL (5′-BODIPY FL-TTGGGGTTATTTTGGGAGGGCGGGGGTT-3′) and BV42-BDPFL (5′-Bodipy FL-AACGCTCACTCCCCCAAGAAGAACCCCCCCCCCCC-CCCCCCCCCCAGTGAGCGTT) from Lumiprobe (Moscow, Russia).

Influenza viruses and allantoic fluid were provided by the Chumakov Federal Scientific Center for Research and Development of Immune and Biological Products of the Russian Academy of Sciences. The following influenza strains were studied: A/chicken/Kurgan/3654-at/2005 (H5N1, influenza A virus) and B/Victoria/2/1987 (influenza B virus). Virus stocks were propagated in the allantoic cavity of 10-day-old embryonated specific pathogen free chicken eggs. Eggs were incubated at 37 °C, cooled at 4 °C 48 h post-infection and harvested 16 h later. The study design was approved by the Ethics Committee of the Chumakov Institute of Poliomyelitis and Viral Encephalitides, Moscow, Russia (Approval #4 from 2 December 2014). Viruses were inactivated via the addition of 0.05% (*v*/*v*) glutaric aldehyde, preserved via the addition of 0.03% (*w*/*v*) NaN_3_ and stored at +4 °C.

### 2.1. Aptamer Preparation

Aptamers are to be properly folded to exhibit the functional activity. The aptamer RHA0385-SH was folded at 2 µM concentration in 10 mM KCl. Aptamers RHA0385-BDPFL and BV42-BDPFL were folded at 2 µM concentration in 10 mM KCl and 140 mM NaCl. The folding process was carried out in the following way: the solution was heated at 95 °C for 5 min and cooled at room temperature.

### 2.2. Preparation and Characterization of Citrate-Stabilized Silver Nanoparticles (AgNP-Citr)

The AgNO_3_ solution (10 mM, 3 mL) was mixed with sodium citrate solution (19 mM, 29 mL). This solution was mixed with fresh solution of NaBH_4_ (26 mM, 34 µL). The reaction mixture was mixed for 30 min using a magnetic stirrer. These nanoparticles (AgNP-Citr) were characterized by several techniques.

The absorption spectrum of AgNP-Citr peaked with a maximum at 396 nm and a width at half-height of 68 nm (Figure S1). AgNP-Citr had a mean diameter of 5 nm (Figure S2A); the AgNP-Citr diameter was estimated by dynamic light scattering using Zetasizer Nano ZS with a He-Ne laser with a wavelength of 633 nm (Malvern Instruments, Worcestershire, UK). The diameter was determined independently using transmission electron microscope Leo 912 AB Omega (Leo, Oberkochen, Germany) with accelerating voltage 100 kV. One to two microliters of AgNP-Citr were applied onto formvar-treated copper mesh with 3 mm diameter. The estimated AgNP diameter was 4 nm.

ZetasizerNano ZS (Malvern, Worcestershire, UK) was used to characterize ζ-potential that was −40±2 mV (Figure S2B). This value corresponds to AgNP-Citr that are stable against spontaneous aggregation. The state of Ag in nanoparticles was estimated using X-ray phase diffractometer Dron-3 (NPP Burevestnik, Saint Petersburg, Russia) with CuKα (λ = 1.54 Ǻ) anode emission. The peaks corresponded to Ag crystal lattice (Figure S3).

### 2.3. Preparation of Silver Nanoparticles According to Leopold and Lendl (AgNP-LL)

Silver nanoparticles (AgNP-LL) were prepared according to the technique described earlier by Leopold and Lendl [21]. One hundred eighty microliters of 167 mM solution of NH_2_OH∙HCl and 120 µL of 500 mM solution of NaOH were mixed with 18.7 mL of water. Then 1 mL of 20 mM AgNO_3_ solution was added. The reaction mixture was mixed for 1 h by a magnetic stirrer at room temperature. AgNP-LL with citrate were obtained by treatment of 12 mL of AgNP-LL with 80 µL of 65 mM solution of sodium citrate. Both types of AgNP were used 3–4 days after preparation. The mean size of these AgNP and their modifications with RHA0385-SH and citrate was nearly 10 nm according to dynamic light scattering performed using ZetasizerNano ZS (Malvern, Worcestershire, UK) (Figure S4A,B). The ζ-potential was −44 ± 6 mV (Figure S4C), as measured by ZetasizerNano ZS (Malvern, Worcestershire, UK).

### 2.4. Characterization of Influenza Viruses

The functional activity of the influenza viruses was estimated by hemagglutination assay. One hundred microliters of 0.5% chicken red blood cells in 140 mM NaCl were placed in V-bottom 96-well plates (Greiner, Kremsmünster, Austria). Viruses were aliquoted with 2-fold dilution (the final volume of mixture was 200 µL). Hemagglutination was estimated after 30 min of incubation. The absence of red dots was interpreted as hemagglutination. The sample with minimal viral content that caused hemagglutination was assigned to contain 1 HAU (hemagglutination unit) viral titer. An example of the hemagglutination assay for an influenza A virus is shown in Figure S5. Viral loads in viral particles per mL (VP/mL) were estimated from hemagglutination units (HAU/mL) based on correlations published earlier (1 HAU ~ 5 × 10^7^) [22].

### 2.5. Determination of Influenza Viruses Using Different Nanoparticles

AgNP-Citr. AgNP-Citr were incubated with 20 nM solution of RHA0385-SH for 1 h at 37 °C. Then, 480 µL of modified AgNP were mixed with 5 µL of 2 µM solution of RHA0385-BDPFL, 15 µL of 5 M solution of NaCl and 250 µL of influenza A virus (or influenza B virus, or allantoic fluid) diluted in 15 mM PBSK (15 mM phosphate buffer pH 7.2, 150 mM NaCl, 15 mM KCl). The mixture was incubated for 10 min; then, SERS spectra were recorded for 200 ms with 25 repeats using handheld Raman analyzer RaPort (Enhanced Spectrometry, San Jose, CA, USA) with a laser excitation wavelength of 532 nm and a power of 30 mW.The experimental series had the following differences in setup:

(1a) The viral load was increased sequentially in the same AgNP sample. This setup was used in this series only.
(1b) Different viral loads were achieved in different AgNP samples. Other details were the same as in 1a series.
(1c) The setup was the same as in 1b series except for the type of aptamer. BV42-BDPFL was used instead of RHA0385-BDPFL.

AgNP-LL treated with a thiol-modified aptamer and citrate. AgNP were incubated with 40 nM solution of RHA0385-SH for 1 h at 37 °C. The resulted AgNP were modified with sodium citrate solution for 2 h (the final citrate concentration was 450 µM). Then, 196 µL of modified AgNP was mixed with 4 µL of 2 µM solution of RHA0385-BDPFL, 50 µL of 5× concentrated PBS and 250 µL of influenza A or influenza B viruses diluted in 10 mM PBSK (10 mM phosphate buffer pH 7.2, 150 mM NaCl, 20 mM KCl). The mixture was incubated for 10 min; then SERS spectra were recorded for 400 ms with 25 repeats using handheld Raman analyzer RaPort (Enhanced Spectrometry, San Jose, CA, USA) with a laser excitation wavelength of 532 nm and a power of 30 mW. This series is marked as 2a.

AgNP-LL treated with citrate. One hundred ninety-six microliters of AgNP-LL was mixed with 4 µL of 2 µM solution of RHA0385-BDPFL, 50 µL of 5× concentrated PBS and 250 µL of influenza A or influenza B viruses diluted in 10 mM PBSK (10 mM phosphate buffer pH 7.2, 150 mM NaCl, 20 mM KCl). The mixture was incubated for 10 min; then SERS spectra were recorded for 400 ms with 25 repeats using handheld Raman analyzer RaPort (Enhanced Spectrometry, San Jose, CA, USA) with a laser excitation wavelength of 532 nm and a power of 30 mW. The differences were as follows:

(2b) AgNP were incubated with 40 nM solution of RHA0385-SH for 1 h at 37 °C before the experiment described above.
(2c) The setup without any modifications was used.

A brief description of all experimental series is provided in Table 1.

## 3. Results

### 3.1. Setup of Aptasensors for Influenza Virus Determination

Colloidal AgNP were tested as a basis for creation of aptasensor for virus determination. Two types of DNA aptamers to influenza hemagglutinin were used. Aptamer RHA0385 [23] was shown to recognize a wide variety of influenza A strains [17,18]; aptamer BV42 [24] had the same ability (unpublished data). The setup of the aptasensor was similar to our previous solid-state aptasensor [17]. It consumed primary aptamers for functionalization of silver and secondary aptamers to provide an analytical signal.

AgNP were functionalized with thiol-modified aptamer RHA0385. Then AgNP were mixed with aptamers conjugated to SERS-active label and were aggregated by increasing ionic strength. Viral particles of A/chicken/Kurgan/3654-at/2005 (H5N1, influenza A virus) and B/Victoria/2/1987 (influenza B virus) were added to the AgNP aggregates in different content.

**Figure 1:**
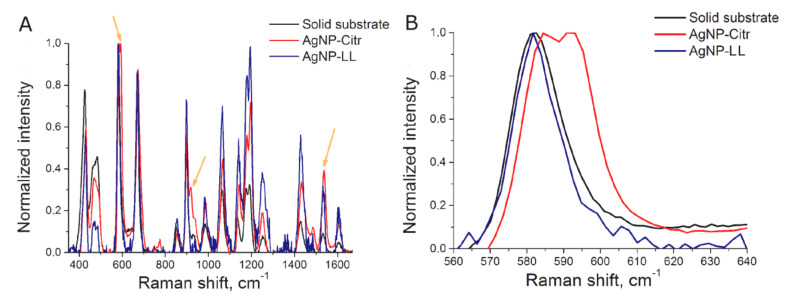
Surface-enhanced Raman spectroscopy (SERS) spectra of BODIPY FL dye onto solid substrate, AgNP-Citr aggregates and AgNP-LL aggregates (**A**). The peaks used for further consideration (**B**).

BODIPY FL was chosen as SERS-active label due to low fluorescence and high SERS intensity. AgNP aggregates provide SERS spectra of BODIPY FL (Figure 1A) that is similar to SERS spectrum of BODIPY FL onto solid-state substrate [17]. The position of peaks was the same both in colloidal and solid-state sensors, but the ratio of intensities at small and large Raman shifts was different for solid-state substrates and colloidal SERS substrates. (Figure 1A). This is due to the different plasmon absorption contours of colloidal silver particles and solid-state SERS substrates.

The spectrum of the BODIPY FL-labeled aptamer onto AgNP-Citr aggregates had several additional peaks compared to AgNP-LL aggregates and solid-state sensors (Figure 1A, Table S1); the peak with the highest intensity is shown in Figure 1B (the peak at 591 cm^−1^). This peak was attributed to BODIPY FL, as it was absent in the spectra of AgNP alone and AgNP with the thiol-modified aptamer (Figure S6). SERS spectra of the same substance differ on various AgNP if the substance forms different complexes with the surface. For example, peaks of citrate are different depending on the AgNP parameters [25,26]. Indeed, SERS spectra of citrate onto AgNP-LL aggregates did not match completely with previously published spectra (Figure S6C). One of the possible explanations for 591 cm^−1^ peak emergence is the following: AgNP-Citr had an extremely small size that is comparable with the size of the aptamer; if one molecule of the BODIPY FL-modified aptamer is trapped between several small AgNP, polarizability of some chemical bond is changed providing a new peak in the spectrum. The SERS signal intensities at 585 cm^−1^ and 595 cm^−1^ (BODIPY FL) were used to estimate the efficiency of aptasensors with AgNP-Citr. The value 595 cm^−1^ for the peak at 591 cm^−1^ was chosen to minimize the contribution of the nearby peak at 585 cm^−1^. The peaks at 585 cm^−1^ (BODIPY FL) and 611 cm^−1^ (citrate peak) were used to estimate the efficiency of aptasensors with AgNP-LL. It should be noted that the majority of modern Raman spectrometers have sufficient resolution to distinguish nearby peaks at 585 cm^−1^ and 591 cm^−1^. For example, RaPort spectrometer has resolution of 4 cm^−1^ in this part of the spectral range.

**Figure 2:**
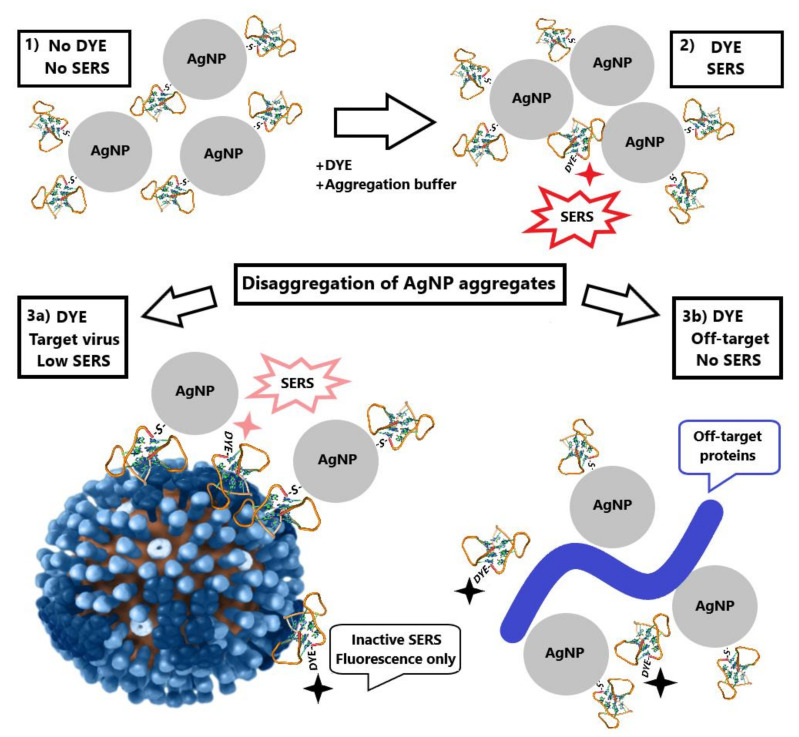
Schematic representation of aptasensor setup. Aptamer-functionalized AgNP (1) were mixed with a labeled aptamer in buffered saline providing AgNP aggregates (2). The aggregates were mixed with target viruses (3a) resulting in weaker SERS signals or with off-target biologicals (3b) losing SERS effect due to the elimination of the labeled aptamer from AgNP aggregates. The picture for influenza A virus was derived from the Public Health Image Library (ID:19013).

Several schemes for aptasensor setup were used. Generally, AgNP were functionalized with a thiol-modified aptamer, mixed with a labeled aptamer in buffered saline. The resulted aggregates were mixed with viruses, and SERS spectra were measured (Figure 2). Specific interaction with the virus provided higher SERS signals than non-specific interactions with components of allantoic fluid or influenza B virus. A detailed description of the experiments is provided in the Materials and Methods section and summarized in Table 1. SERS signal from BODIPY FL appeared only when the dye was trapped between several AgNP. In other cases, only high fluorescence was detected.

### 3.2. Determination of Influenza Virus with AgNP-Citr

**Figure 3:**
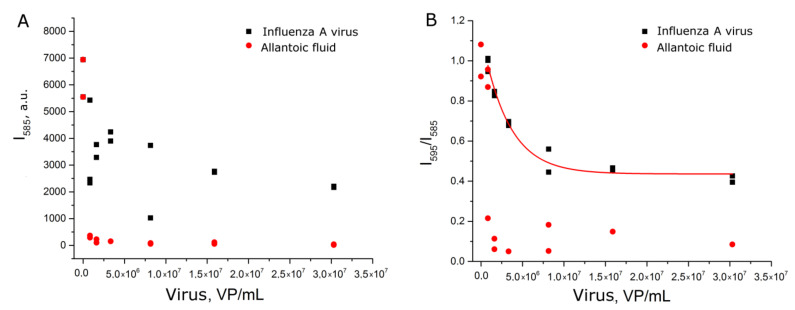
Concentration dependencies of absolute SERS signal intensity of the label (**A**) and relative SERS signal intensity of BODIPY FL peaks (**B**) for influenza virus and allantoic fluid. Allantoic fluid was diluted identically to influenza A virus.

Firstly, we performed subsequent addition of aliquots of the virus to the same samples of AgNP-Citr (setup 1a). Allantoic fluid decreased drastically the intensity of SERS signal due to non-specific interactions with AgNP, whereas samples with influenza A viruses had slightly diminished signals. As the quantity of AgNP was the same in subsequent samples, monotonous dependence was expected. However, the considerable scatter of points was observed for BODIPY FL peaks (Figure 3A and Figure S7). Next, we used relative SERS signal intensities; namely, the newly emerged peak (intensity at 595 cm^−1^ was used) was normalized to the 585 cm^−1^ peak from the label. The dependence became much more smooth (Figure 3B and Figure S8).

The dynamic range of the curve was between 1 × 10^6^ and 1 × 10^7^ VP/mL (Figure S9); the range for reliable quantitative determination of influenza A virus was 1 × 10^6^–3 × 10^7^ VP/mL (the upper limit of detection was not reached in the series). Here the peak from the labeled aptamer trapped between several small AgNP was normalized to the regular BODIPY FL peak. The resulting relative value was used as an analytical signal; the procedure diminished the low reproducibility of aggregation.

**Figure 4:**
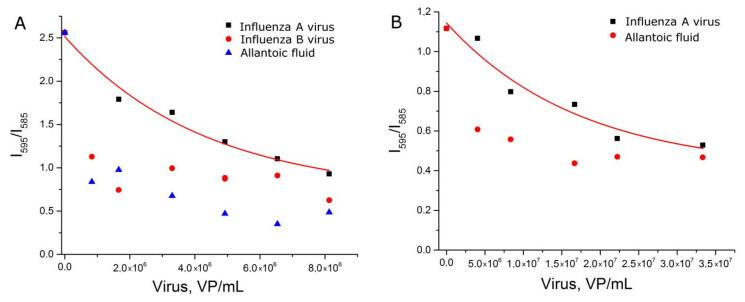
Concentration dependencies of relative SERS signal intensities of the labeled aptamer in experimental setups 1b (**A**) and 1c (**B**). Allantoic fluid was diluted identically to influenza A virus. Fittings with exponential functions are provided (see Table S2 for details).

The experiments with setup 1b reproduced setup 1a, but each sample was prepared independently. Nevertheless, the dependence of relative SERS signal intensity from the viral load was reproduced (Figure 4A). Samples with influenza A virus had significantly higher signal intensity than allantoic fluid and off-target influenza B virus. The reproducibility of the curves can be illustrated by the match within the error of curvatures (Table S2).

Next, we tested another BODIPY FL-labeled aptamer to hemagglutinin. Aptamer BV42 has putative i-motif structure [27] and inhibits hemagglutinin function with IC_50_ = 8 nM [24]. Its inhibitory activity is comparable to activity of the RHA0385 aptamer [28]. Thiol-modified RHA0385 and BODIPY FL-labeled BV42 aptamers were used in the experimental setup 1c. Replacement of the aptamer decreased discrepancy between samples with influenza A virus and control samples (Figure 4B). The exponential fit of the curve differed from those of setups 1b and 1c (Table S2). The dynamic range (5 × 10^6^–2 × 10^7^ VP/mL) was narrower and shifted to higher viral loads compared to the setup with BODIPY FL-labeled RHA0385. Thus, the type of aptamer affects the aptasensor characteristics.

### 3.3. Determination of Influenza Virus with AgNP-LL

One more AgNP type was tested. AgNP were optimized exactly for SERS measurements by Leopold and Lendl [21], having the mean particle diameter of 10 nm (Figure S4). AgNP-LL were incubated with citrate in order to imitate the surface of AgNP-Citr.

**Figure 5:**
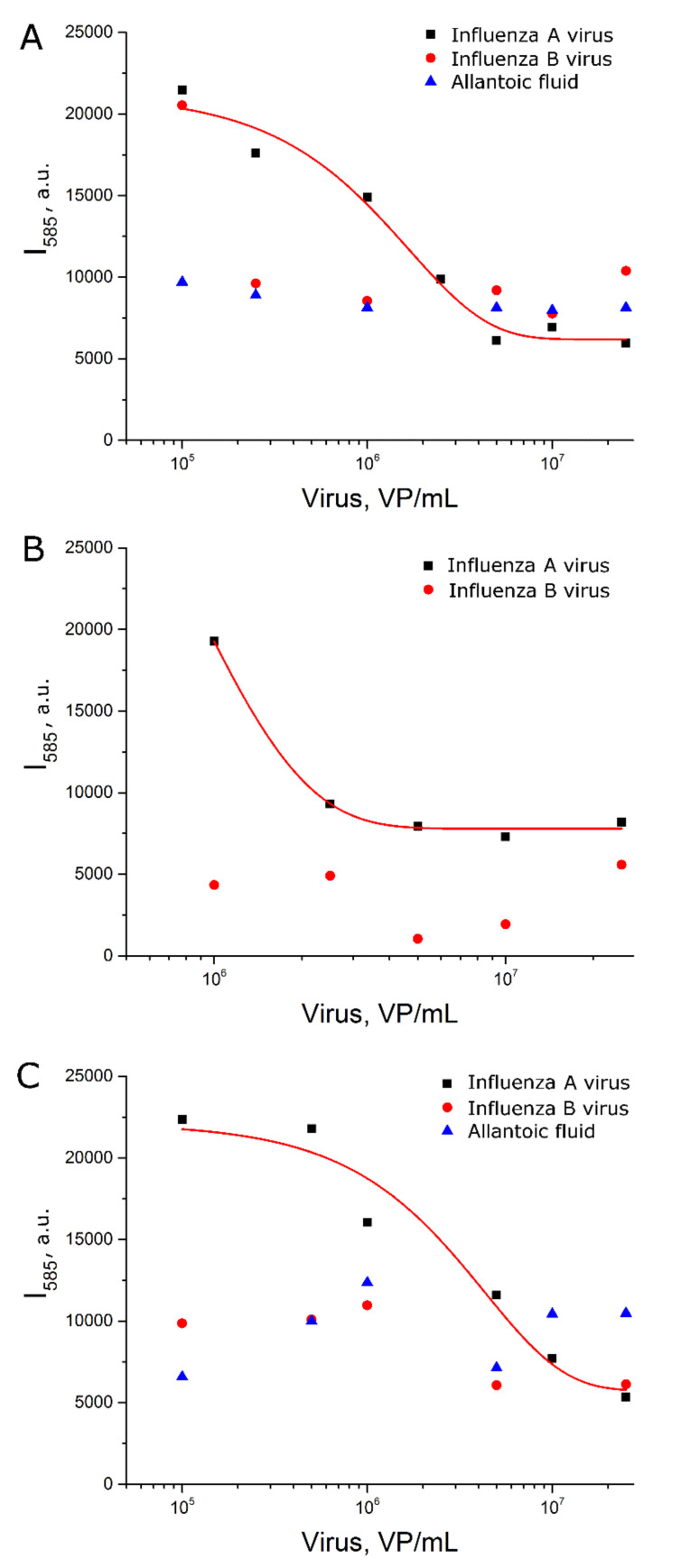
Concentration dependencies of SERS signal intensity of BODIPY FL for experimental setup 2a (**A**), 2b (**B**) and 2c (**C**). Allantoic fluid was diluted identically to influenza A virus. Fittings with exponential functions are provided (see Table S2 for details).

First, AgNP-LL were functionalized with a thiol-modified aptamer with subsequent incubation in a sodium citrate solution (setup 2a). These AgNP provided peaks of citrate spectrum (Figure S6) that were supposed to be useful as an internal standard. However, the relative peak intensity had no dependence on virus content (Figure S10). Absolute values of SERS signal intensity of BODIPY FL were revealed to be a relevant parameter which was dependent on influenza virus content (Figure 5A). The dynamic range of the curve was between 2 × 10^5^ and 2 × 10^6^ VP/mL, which corresponds to the highest sensitivity among the different setups.

Second, the order of AgNP modification was changed. AgNP-LL were incubated with citrate and subsequently functionalized with the thiol-modified aptamer (setup 2b). The dynamic range was extremely narrow (Figure 5B). This setup cannot be used for virus quantification.

Third, AgNP-LL were incubated with citrate without further functionalization with the aptamer (setup 2c). In this case, the curve was of a good quality with the dynamic range between 6 × 10^5^ and 3 × 10^6^ VP/mL. This setup was less efficient compared to setup 2a, which illuminates the role of functionalization of AgNP with aptamers for assay sensitivity.

## 4. Discussion

A wide variety of SERS-based techniques was developed to detect viruses. Most of them consumed antisense oligonucleotides (ASO) to capture viral genomes. This field has been reviewed in detail in our recent work [3]. In general, ASO-based techniques have lower limits of detection if the virus genome is DNA because of higher stability of DNA duplexes compared to RNA/DNA duplexes [29,30]. Influenza has an RNA genome; and a comparison between assays with genome capture and surface protein capture revealed the latter ones to be an unambiguously better choice for highly sensitive assays [12,17,30]. Surface proteins can be captured by either antibodies or aptamers. Aptamers have advantages due to a relatively small size and a variety of modifications that can be introduced site-specifically during chemical synthesis [3].

SERS-based assays with the highest performance consume solid nanostructured substrates with complex surfaces [3]. For example, aptamer-covered multilayer substrates made up of polyethylene naphthalate, gold, perfluorodecanethiol and one more gold layer were used to detect influenza A virus with a limit of detection of nearly 100 pfu/mL (10^4^ VP/mL [30]) [13]. This technique was shown to be useful for virus quantitation, as opposed to another aptamer-based technique with a similar limit of detection that was useful for qualitative analysis only [17]. Both techniques consume solid substrates made up using advanced equipment to provide metal deposition in a controlled and reproducible manner.

Contrary to solid substrates, nanoparticles can be synthesized in a “one pot” manner at room temperature. Many techniques have been described that provide good reproducibility of AgNP size, shape and ζ-potential [21,31,32]. Here we used two types of AgNP with simple formulations: the first one was citrate stabilized with a mean diameter of 4 nm; the second one was chloride stabilized with mean diameter 10 nm. The assay setup comprised aggregation of aptamer-functionalized AgNP in the presence of a labeled aptamer with subsequent disruption of aggregates by viruses and other biological molecules. The efficiency of disintegration of AgNP aggregates differed in the presence of aptamer-targeted and aptamer-off-targeted viruses. Supposed mechanisms include AgNP organization onto the viral particles with preservation of SERS signal and the removal of the labeled aptamer from proximity to AgNP due to non-specific interactions of AgNP with biological molecules (Figure 2).

Absolute SERS signal intensity of the label was successfully used as an analytical signal in the case of AgNP-LL but not AgNP-Citr. In the case of AgNP-Citr, the relative SERS signal can be used as a reproducible analytical signal. Relative intensity of the specific peak from the complex of the BODIPY FL-modified aptamer with AgNP-Citr was used. Non-specific interactions of biological molecules with AgNP surface with partial destruction of the complex between the BODIPY FL-modified aptamer and AgNP-Citr citrate ions can be proposed as a possible mechanism that explains the higher rate of decrease of the analytical signal. It is interesting to study the polarization of peaks at 585 cm^−1^ and 591 cm^−1^ of BODIPY FL in order to increase the dynamic range for detection of the influenza virus. The particular Raman peak could be amplified by excitation with parallel polarization or cross-polarization [33].

Usage of an internal standard was shown to be a potent approach for a wide range of analytes including ions, low-molecular compounds, exosomes and whole bacterial cells [34,35,36,37,38]. Some approaches utilize a signal from molecules entrapped in AgNP as a standard [34,38]. If the analyte is a cell, several membrane components can be used to normalize the signal from the label, enhancing accuracy and reproducibility of the assay [35]. In other approaches, several types of AgNP were used simultaneously; some of AgNP were used as an internal standard for other target-bound AgNP [36]. Our assay with AgNP-Citr did not require additional compound due to peculiarities of the interaction between labeled the aptamer and these particular types of AgNP.

AgNP-LL provided more reproducible SERS signal; and thus, the results for absolute SERS signal intensity on AgNP-LL were nearly of the same quality as relative SERS signal intensity on AgNP-Citr. We tried to use citrate as an internal standard for AgNP-LL, but the reproducibility of citrate adsorption and desorption was very low. Possibly, it was due to a layer of chloride ions adsorbed onto AgNP-LL. Both AgNP-Citr and AgNP-LL preparation techniques are simple and consume affordable chemicals. The simplicity and the low cost make these AgNP very attractive for a practical implementation.

Several SERS-based techniques with lower LOD and wider dynamic range have been described, but all of them consume much more sophisticated procedures for preparation of SERS-active structures and/or sample preparation [39]. For example, antibody-modified AgNP allowed detection of as low as 100 VP/mL with dynamic range over 10^7^ VP/mL, but the sample preparation requires nearly 2 h with usage of adsorption onto nitrocellulose membrane [39]. Another antibody-based assay had a dynamic range over 4 × 10^5^–10^9^ VP/mL with setup duration more than 3.5 h for step-by-step adsorption of the assay components [12]. The aptasensor with similar characteristics allowed determination of 10^4^–10^6^ VP/mL; the experimental setup was rather quick (>10 min) but needed a sophisticated multilayer substrate [13]. Our aptasensor had LOD 2 × 10^5^ VP/mL and a dynamic range of 2 × 10^5^–2 × 10^6^ VP/mL; however, the time of the assay was below 15 min, and the AgNP preparation was very simple.

Considering further development of nanoparticle-based aptasensors for rapid diagnostics, there are several possible ways to improve the setups described. The first way is to reverse the concentration dependence of the analytical signal, i.e., to get the increase of SERS intensity with the increase of virus content. This technique allows the increase of the difference between samples with and without the target; FRET-based techniques are good examples of this approach [40,41]. The second way is to increase the affinity of the aptamers to the virus, which results in better sensitivity of the assay. The example with two different aptamers to hemagglutinin in this article (Figure 4) clearly indicates that affinity is a key determinant. We also speculate that highly affine aptamers could provide broader dynamic range of the aptasensors.

## 5. Conclusions

A new approach was developed combining rapid specific detection and a possibility for quantitative determination of viruses using the example of influenza A virus. Specificity was provided by aptamers to influenza hemagglutinin, whereas sensitivity was provided by a SERS-based technique. The proposed technique can be classified as a rapid diagnostic test due to a short time of analysis (below 15 min) and simple sample preparation (the assay is homogeneous). AgNP with the simplest preparation and high stability were chosen. Absolute and relative SERS signal intensities could be used as an analytical signal. The optimal aptasensor had LOD of 2 × 10^5^ VP/mL and a dynamic range of 2 × 10^5^–2 × 10^6^ VP/mL.

## Figures and Tables

**Table 1:** A brief description of components in different experimental series.

Type of AgNP	AgNP-Citr			AgNP-LL		
Setup	1a	1b	1c	2a	2b	2c
Reagents	Concentrations					
Ag^+^	0.3 mM	0.3 mM	0.3 mM	0.4 mM	0.4 mM	0.4 mM
RHA0385-SH	20 nM	20 nM	20 nM	40 nM	40 nM	-
Citrate	-	-	-	450 µM	450 µM	450 µM
RHA0385-BDPFL	13 nM	13 nM	-	16 nM	16 nM	16 nM
BV42-BDPFL	-	-	13 nM	-	-	-

## Data Availability

The data presented in this study are available on request from the corresponding author. The data are not publicly available due to privacy.

## Supplementary Materials

The following are available online at https://www.mdpi.com/1422-0067/22/4/1842/s1.

## Abbreviations

AgNP-Citr	Citrate stabilized silver nanoparticles
AgNP-LL	Silver nanoparticles synthesized according to Leopold and Lendl
ASO	Antisense oligonucleotides
FRET	Fluorescence resonance energy transfer
HxNy	Abbreviation of influenza subtype, namely, hemagglutinin subtype x (1 or 5) and neuraminidase subtype y (1)
HA	Hemagglutinin
HAU	Hemagglutination unit
HIV	Human immunodeficiency virus
pfu	Plaque forming unit
PCR	Polymerase chain reaction
LFIA	Lateral flow immunochromatographic assays
LOD	Limit of detection
SERS	Surface-enhanced Raman spectroscopy
VP	Viral particle
