# Supplementary material for: SERS-Based Colloidal Aptasensors for Quantitative Determination of Influenza Virus
Source: Int J Mol Sci. 2021 Feb 12;22(4):1842. doi: 10.3390/ijms22041842 (PMC7918581; doi:10.3390/ijms22041842)
Supplement: Supplementary file 1 [file ijms-22-01842-s001.pdf]

SERS-based colloidal aptasensors for quantitative determination of influenza virus

Dmitry Gribanyov[1], Gleb Zhdanov[2], Andrey Olenin[2], Georgii Lisichkin[2], Alexandra Gambaryan[3], Vladimir Kukushkin[1]*, Elena Zavyalova[2]*

[1]Institute of Solid State Physics RAS, Chernogolovka 142432, Russia

[2]Chemistry Department, Lomonosov Moscow State University, Moscow 119991, Russia

[3]Chumakov Federal Scientific Center for Research and Development of Immune and Biological Products RAS, Moscow 108819, Russian Federation

* Correspondence: kukushvi@mail.ru (V.K.); zlenka2006@gmail.com (E.Z.)

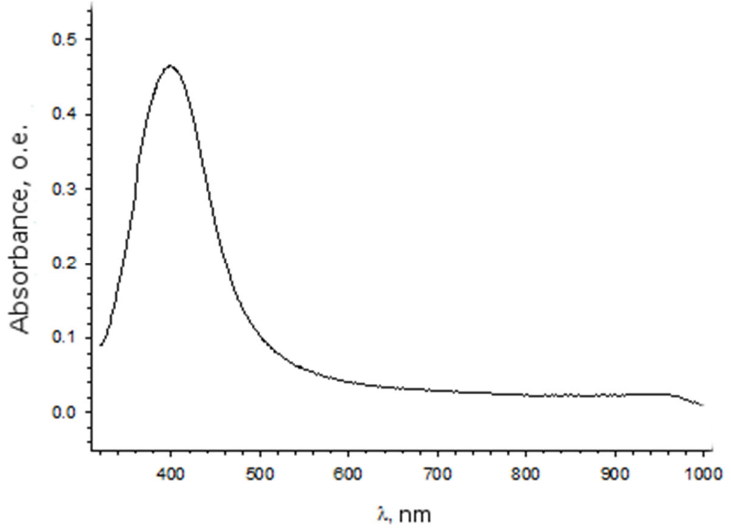

Figure S1. UVI spectrum of AgNP-Citr.

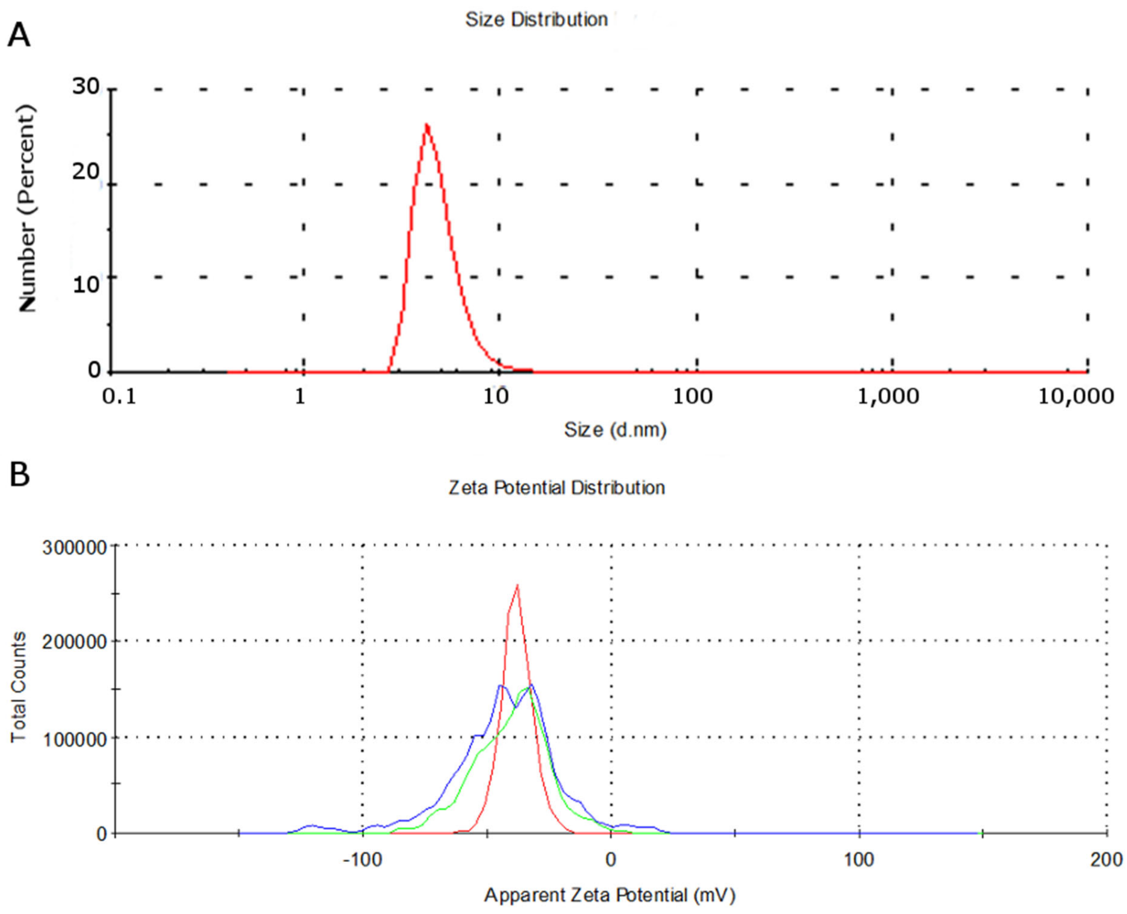

Figure S2. Dynamic light scattering of AgNP-Citr (number distribution, A) and estimation of ζ-potential (B).

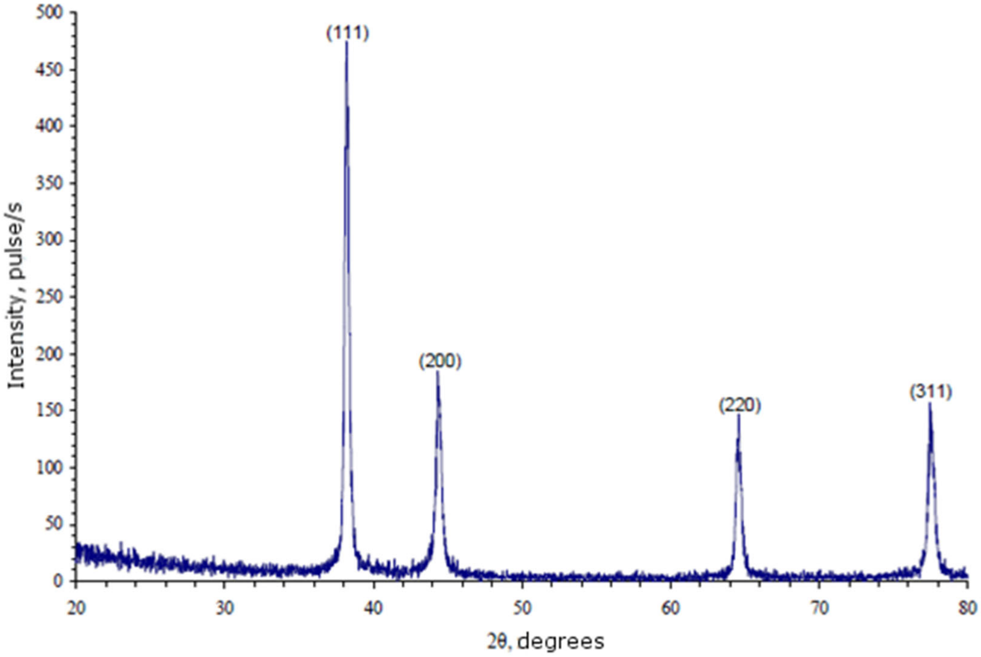

Figure S3. X-ray phase diffractogram of AgNP-Citr; peaks correspond to Ag crystal lattice.

A

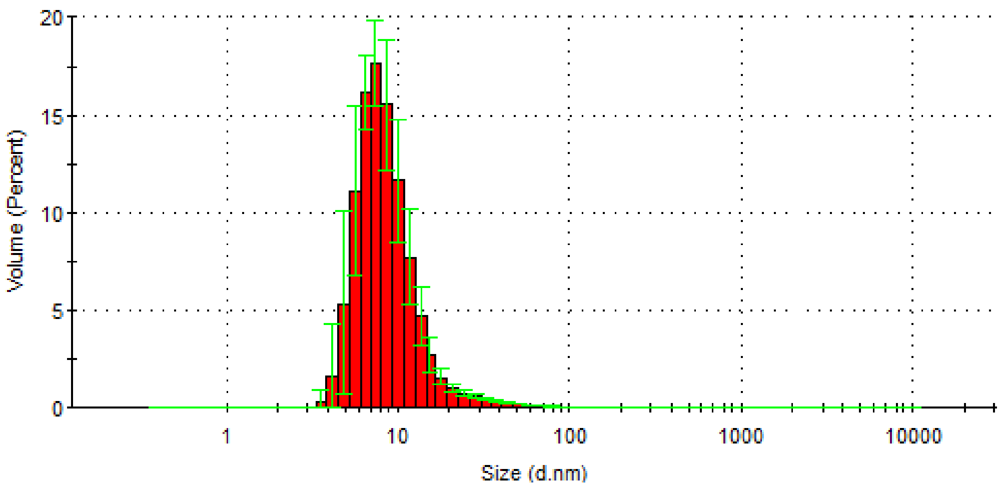

B

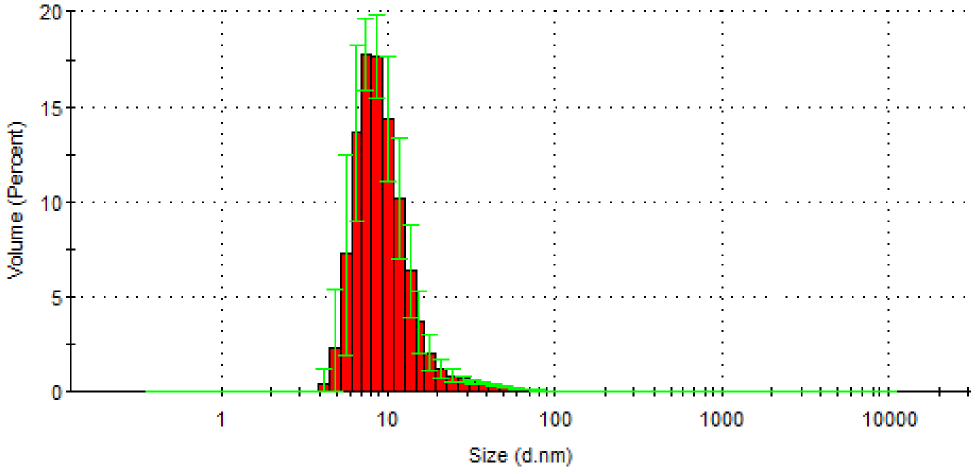

C

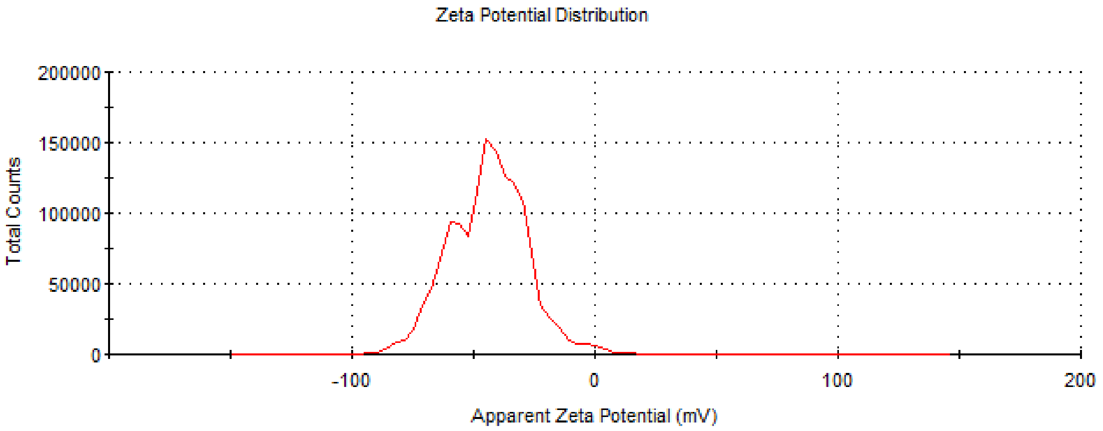

Figure S4. Dynamic light scattering of AgNP-LL: volume distribution of empty AgNP (A) and AgNP with thiol-modified aptamer and citrate (B); estimation of ζ-potential of unmodified AgNP-LL (C).

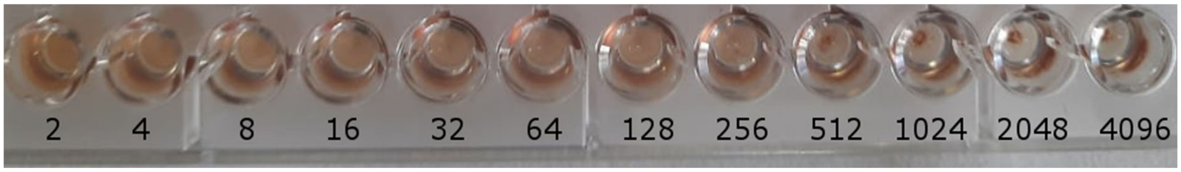

Figure S5. Hemagglutination assay for influenza A virus. Numbers indicate the dilution of virus solution.

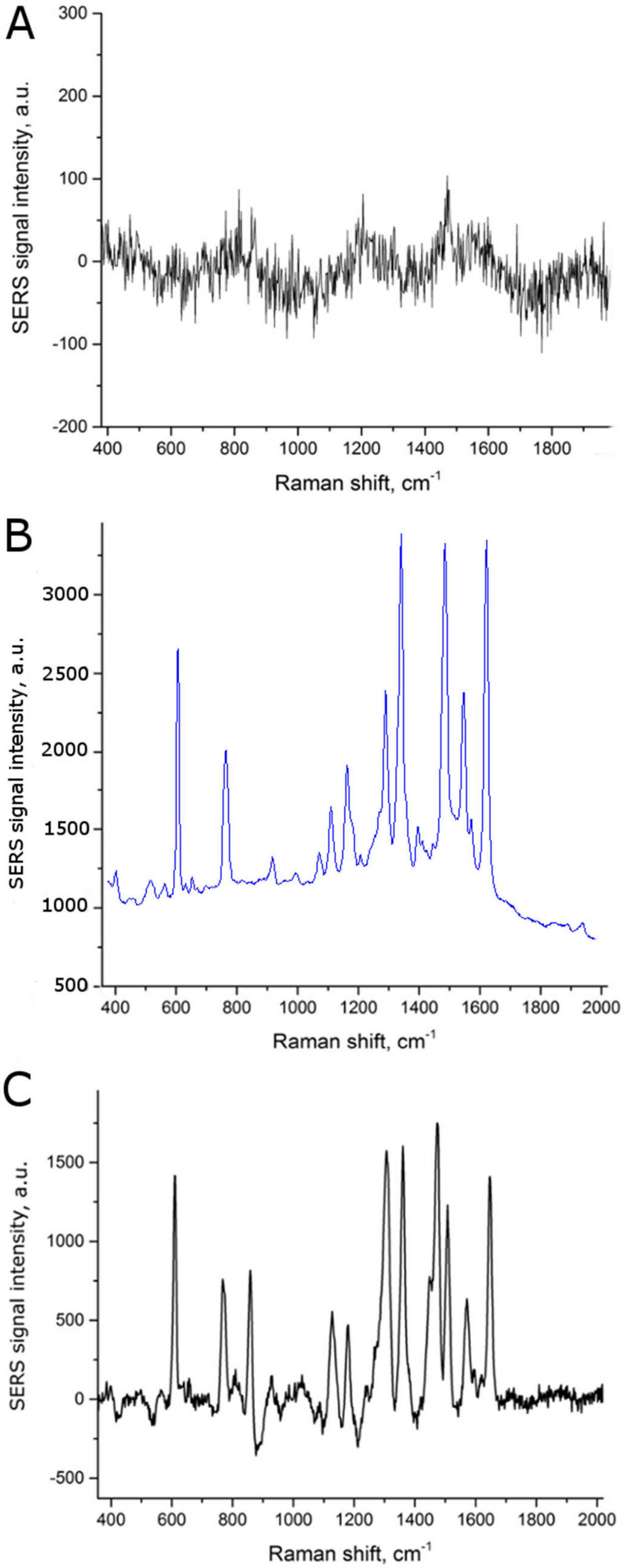

Figure S6. SERS spectra of AgNP-Citr (A), AgNP-LL-Citr with thiol-modified aptamer added during the AgNP synthesis (B) and AgNP-LL with 35 mM sodium citrate (C). All AgNP were aggregated with salt. The spectra (B) and (C) contains peak at 611 cm^{-1} and no peaks in the region 570-600 cm^{-1}.

Table S1. The list of the peaks in Raman spectra from the different setups. s – strong, m – medium, w – weak, br – broad.

1a	1b	1c	2a	2b	2c
428 cm^{-1}, s	428 cm^{-1}, s	428 cm^{-1}, s	426 cm^{-1}, s	426 cm^{-1}, s	426 cm^{-1}, s
467 cm^{-1}, m, br	467 cm^{-1}, m, br	467 cm^{-1}, m, br	467 cm^{-1}, m, br	467 cm^{-1}, m, br	467 cm^{-1}, m, br
542 cm^{-1}, w	542 cm^{-1}, w	542 cm^{-1}, m			
584 cm^{-1}, s	584 cm^{-1}, s	584 cm^{-1}, s	581 cm^{-1}, s	581 cm^{-1}, s	581 cm^{-1}, s
591 cm^{-1}, s	591 cm^{-1}, s	591 cm^{-1}, s			
			610 cm^{-1}, m	611 cm^{-1}, w	611 cm^{-1}, m
672 cm^{-1}, s	672 cm^{-1}, s	672 cm^{-1}, s	670 cm^{-1}, s	670 cm^{-1}, s	670 cm^{-1}, s
772 cm^{-1}, w	772 cm^{-1}, w	772 cm^{-1}, w	767 cm^{-1}, m	769 cm^{-1}, w	768 cm^{-1}, m
		823 cm^{-1}, w			
855 cm^{-1}, w	855 cm^{-1}, w	855 cm^{-1}, w	856 cm^{-1}, m	856 cm^{-1}, m	856 cm^{-1}, m
899 cm^{-1}, s	899 cm^{-1}, s	899 cm^{-1}, s	898 cm^{-1}, s	898 cm^{-1}, s	898 cm^{-1}, s
916 cm^{-1}, m, br	916 cm^{-1}, m, br	916 cm^{-1}, m, br			
			931 cm^{-1}, w	934 cm^{-1}, w	931 cm^{-1}, w
985 cm^{-1}, m	985 cm^{-1}, m	984 cm^{-1}, m	982 cm^{-1}, m	984 cm^{-1}, m	983 cm^{-1}, m
1065 cm^{-1}, s	1065 cm^{-1}, s	1065 cm^{-1}, s	1063 cm^{-1}, s	1063 cm^{-1}, s	1063 cm^{-1}, s
			1081 cm^{-1}, m	1081 cm^{-1}, m	1081 cm^{-1}, w
1104 cm^{-1}, w	1107 cm^{-1}, w	1107 cm^{-1}, w			
1141 cm^{-1}, m	1141 cm^{-1}, m	1141 cm^{-1}, m	1138 cm^{-1}, s	1139 cm^{-1}, s	1138 cm^{-1}, s
1178 cm^{-1}, s	1178 cm^{-1}, s	1178 cm^{-1}, s	1176 cm^{-1}, s	1177 cm^{-1}, s	1176 cm^{-1}, s
1195 cm^{-1}, s	1195 cm^{-1}, s	1195 cm^{-1}, s	1194 cm^{-1}, s	1195 cm^{-1}, s	1194 cm^{-1}, s
1249 cm^{-1}, m	1251 cm^{-1}, m	1250 cm^{-1}, m	1246 cm^{-1}, s	1246 cm^{-1}, s	1248 cm^{-1}, m
			1262 cm^{-1}, m	1262 cm^{-1}, s	1262 cm^{-1}, w
			1308 cm^{-1}, w	1308 cm^{-1}, w	1308 cm^{-1}, w
		1327 cm^{-1}, w, br			
1362 cm^{-1}, w, br	1362 cm^{-1}, w, br	1362 cm^{-1}, w, br	1361 cm^{-1}, s	1361 cm^{-1}, m	1361 cm^{-1}, m
1431 cm^{-1}, m	1431 cm^{-1}, m, br	1431 cm^{-1}, m	1430 cm^{-1}, s	1430 cm^{-1}, s	1430 cm^{-1}, s
			1474 cm^{-1}, w	1474 cm^{-1}, w	1474 cm^{-1}, w
1486 cm^{-1}, w	1486 cm^{-1}, w	1486 cm^{-1}, w			

			1509 cm^{-1}, m	1509 cm^{-1}, w	1509 cm^{-1}, w
1535 cm^{-1}, m	1537 cm^{-1}, m	1537 cm^{-1}, m	1532 cm^{-1}, m	1532 cm^{-1}, m	1532 cm^{-1}, m
			1572 cm^{-1}, w	1572 cm^{-1}, w	1572 cm^{-1}, w
1603 cm^{-1}, w	1603 cm^{-1}, w	1603 cm^{-1}, w	1603 cm^{-1} w	1603 cm^{-1}, w	1603 cm^{-1} w
			1647 cm^{-1}, s	1647 cm^{-1}, m	1647 cm^{-1}, m

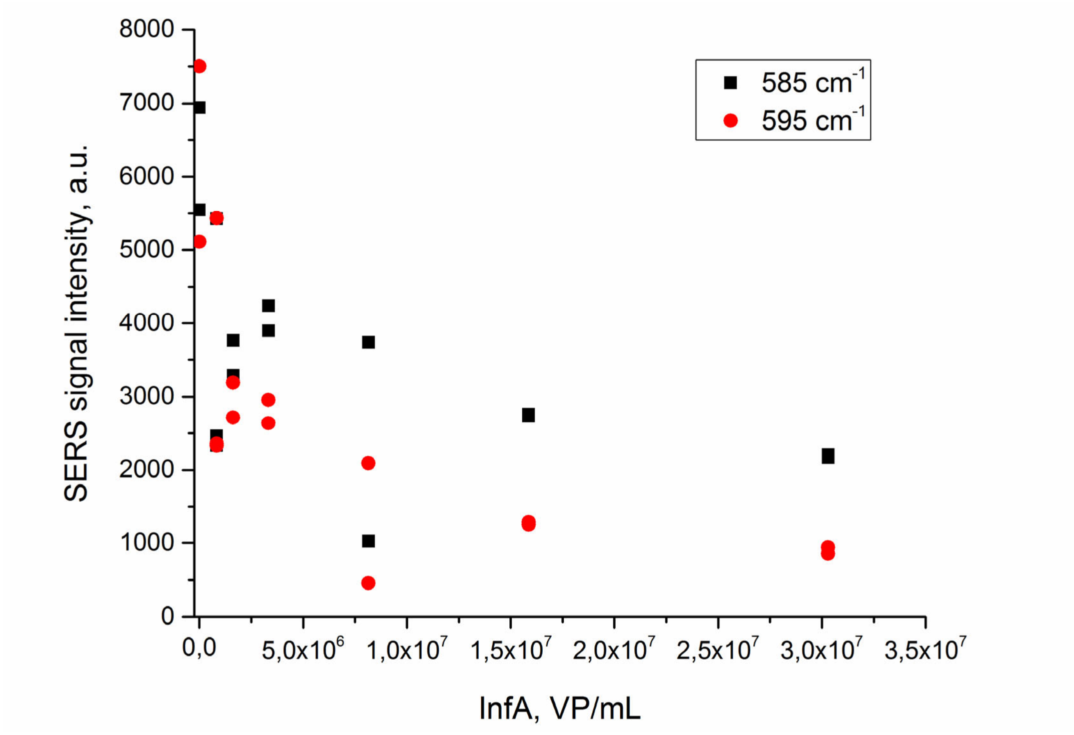

Figure S7. Concentration dependencies of Raman intensities of peaks 585 cm^{-1} and 595 cm^{-1} for the setup 1a.

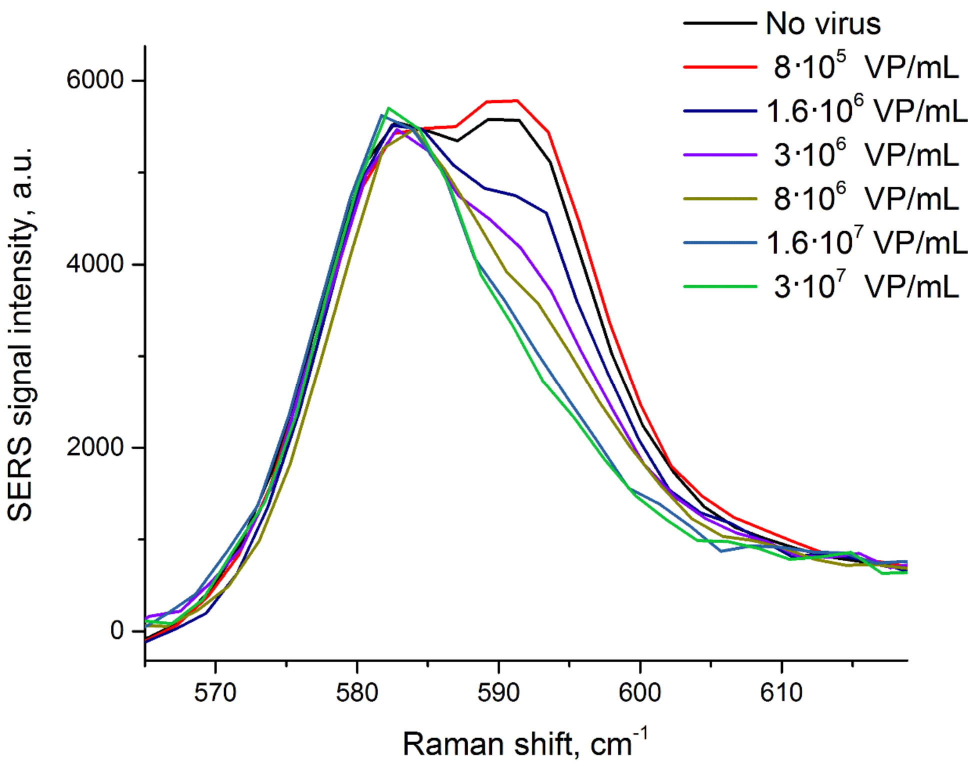

Figure S8. SERS spectra for the aptasensor with setup 1a containing different viral loads of influenza A virus.

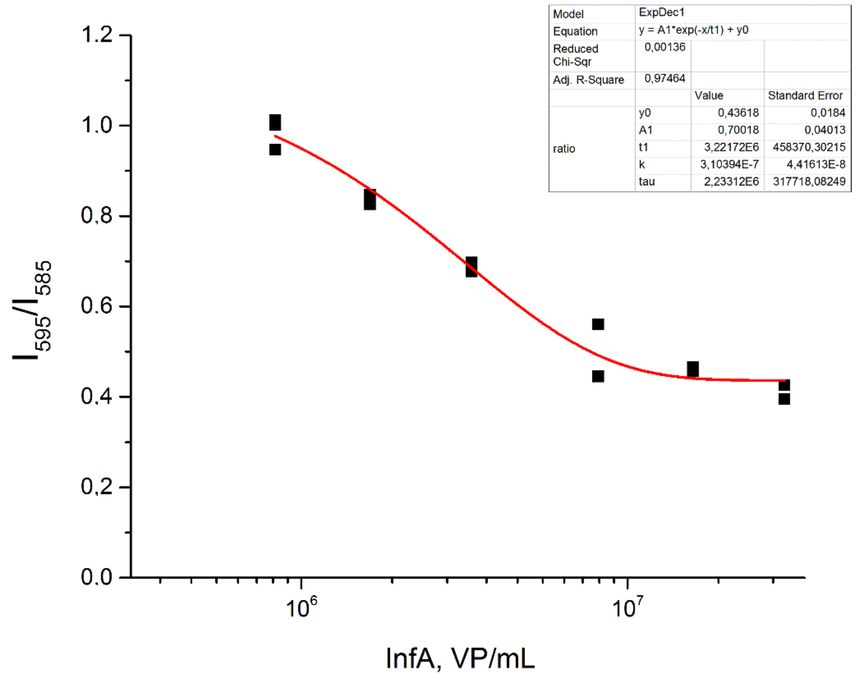

Figure S9. Dependence of the relative Raman signal from influenza A virus content in the sample and its approximation with exponential function. The dynamic range is 10^6-10^7 VP/mL.

Table S2. Parameters of the approximation curves represented in Figures S9 (setup 1a), 4A (setup 1b), 4B (setup 1c), 5A (setup 2a) and 5C (setup 2c). The datasets were approximated with exponential function (y = A1*exp(-x/t1) + y0). Similar values of an inflection point (t1) are colored in green; the highest value is colored in red; the lowest value is colored in blue.

	1a	1b	1c
y0	0.436 ± 0.018	0.7 ± 0.3	0.41 ± 0.19
A1	0.70 ± 0.04	1.8 ± 0.3	0.74 ± 0.18
t1	$3.2 \cdot 10^6 \pm 0.5 \cdot 10^6$	$4.3 \cdot 10^6 \pm 1.5 \cdot 10^6$	$1.7 \cdot 10^7 \pm 0.9 \cdot 10^7$
Reduced Chi-Sqr	0.0014	0.011	0.005
Adj. R-Square	0.97	0.97	0.92

	2a	2c	
y0	$6.2 \cdot 10^3 \pm 0.7 \cdot 10^3$	$5.7 \cdot 10^3 \pm 1.8 \cdot 10^3$	
A1	$1.50 \cdot 10^4 \pm 0.11 \cdot 10^4$	$1.6 \cdot 10^4 \pm 0.2 \cdot 10^4$	
t1	$1.7 \cdot 10^6 \pm 0.4 \cdot 10^6$	$4.3 \cdot 10^6 \pm 1.7 \cdot 10^6$	
Reduced Chi-Sqr	$1.3 \cdot 10^6$	$3.5 \cdot 10^6$	
Adj. R-Square	0.97	0.93	

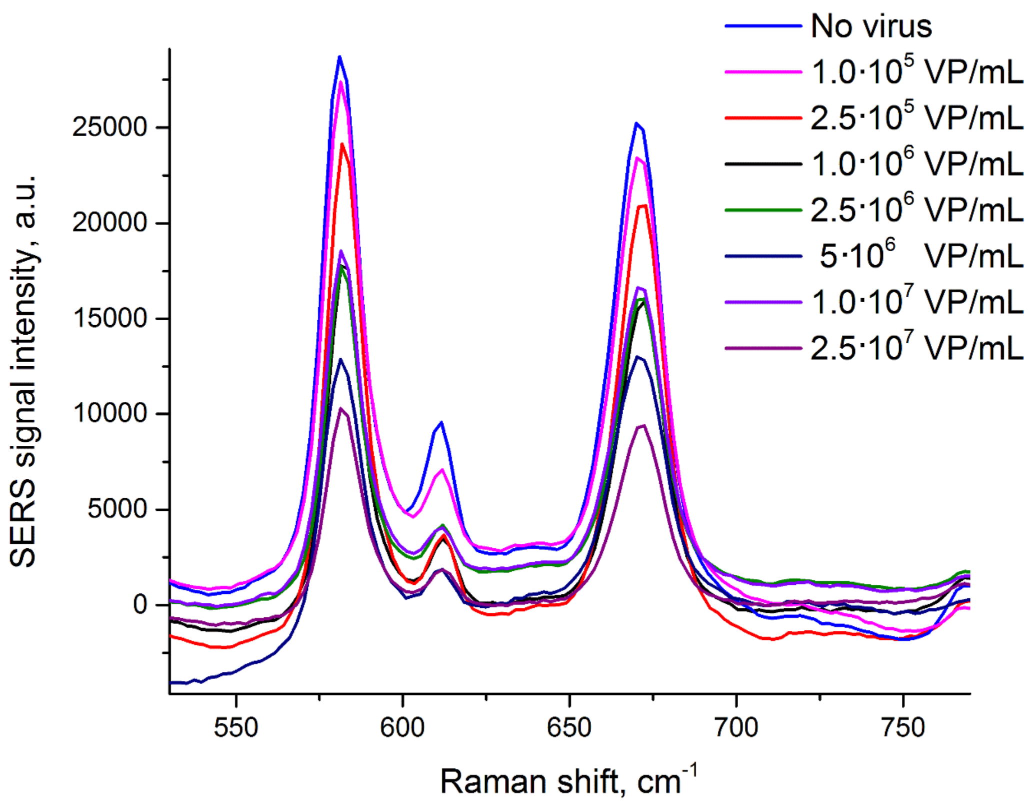

Figure S109. Raman spectra of the samples with AgNP-LL (setup 2a) and different influenza A virus content.
